# Relationship between Investigative Biomarkers and Radiographic Grading in Patients with Knee Osteoarthritis

**DOI:** 10.1155/2009/747432

**Published:** 2009-04-06

**Authors:** Eduardo Anitua, Mikel Sánchez, Maria de la Fuente, Juan Azofra, Mar Zalduendo, Jose J. Aguirre, Isabel Andía

**Affiliations:** ^1^Biotechnology Institute (BTI IMASD), PRGF Research Department, c/ Jacinto Quincoces sn, 01007 Vitoria, Spain; ^2^UCA “Mikel Sanchez”, USP-La Esperanza Clinic, c/La Esperanza 3, 01002 Vitoria, Spain

## Abstract

*Objective*. To examine new investigative biomarkers and their relevance for radiographic severity in knee osteoarthritis. *Methods*. The group comprised 63 patients with 73 knees examined. Patients were divided according to radiographic severity to allow for comparison of biomarker levels. Hyaluronic acid (HA), matrix metalloproteases (MMP-1, MMP-3 and MMP-13), tissue inhibitors of metalloproteases (TIMP-1 and TIMP-2), platelet-derived growth factor (PDGF-AB), transformed growth factor (TGF-*β*), vascular endothelial growth factor (VEGF), hepatocyte growth factor (HGF) and insulin-like growth factor (IGF-I) were measured on synovial fluid and in plasma releasate at a single time point. Principal component analysis (PCA) followed by analysis of covariance were applied to evaluate data. *Results*. Four different groups of biomarker were identified in plasma releasates. The first (platelet number, PDGF-AB and TGF-*β*) and second groups (HA and IGF-I) were related to radiographic severity, *P* = .005 and *P* = .022, respectively. The third (MMP-1 and TIMP-2) and fourth groups (MMP-3 and TIMP-1) represented the catabolic balance, but were not associated to radiographic grading. Three different clusters of biomarkers were found in synovial fluid but did not show any significant association to radiographic grading. *Conclusions*. New imaging approaches to assess structural deterioration and correlation with biomarker levels are warranted to advance in OA research.

## 1. Introduction

Despite the vast increase in molecular knowledge accrued during the last few years, a major breakthrough in OA therapy has not emerged [1]. Limiting factors in current efforts are somewhat attributed to the poor understanding of the molecular basis of disease progression and the lack of dynamic biomarkers that reflect specific biological or pathological processes [2].

Accordingly, a number of studies have focused on finding objective tissue-specific indicators of pathogenic processes in both synovial and peripheral fluids. A major breakthrough in this research was the finding of panels of biomarkers released into urine and serum specifically reflecting the breakdown of major cartilage macromolecules and bone turnover [3]. Other investigative biomarkers reflecting degradative mechanisms (MMPs and TIMPs) of cartilage or representing essential cell-to-cell or cell-to-matrix signaling (Growth Factors, GFs) in serum or plasma are also under examination [4, 5].

The present cross-sectional study was undertaken in order to contrast the levels of GFs in addition to hyaluronic acid (HA), matrix metalloproteases (MMP-1, MMP-3, MMP-13), tissue inhibitors of metalloproteases (TIMP-1 and TIMP-2) in both the Preparation Rich in Growth Factor (PRGF) releasate and synovial fluid (SF) from patients with OA. We hypothesized that the composition of plasma releasate and/or SF may influence radiographic severity of OA. Therefore, patients were divided according to radiographic severity to allow for comparison of biomarker level.

## 2. Methods

The local Ethics Committee approved the study and all patients signed a detailed informed consent form. The study was conducted with 63 consecutive patients with clinical and radiographic evidence of knee OA, according to the ACR criteria, [6] and with joint effusion detected clinically. These patients were a subset of a larger prospective clinical study aiming to evaluate the efficacy of PRGF for the treatment of knee OA. Idiopathic but not secondary posttraumatic or inflammatory OA were included. Patients with generalized OA or arthroscopic lavage in the year previous to treatment, or intra-articular treatment within the previous three months were excluded. Anterior-posterior weight bearing radiographs were scored for Ahlbäck radiographic severity grade by two trained observers in consensus.

### 2.1. Blood and Synovial Fluid Sampling

To obtain PRGF from the patients, fasting venous blood was withdrawn into 9 mL tubes containing 3.8% (wt/volt) sodium citrate. PRGF was prepared by centrifugation at 640 g for 8 minutes. The platelet count in peripheral blood and PRGF was determined using the hematological analyzer MICROS 60 from ABX (Abingdon, UK). Plasma releasates were obtained after plasma coagulation with calcium at a final concentration of 22.8 mM followed by 1 hour incubation at 37°C. Longer times of incubation did not change the releasate composition.

All patients presented joint effusion, 11.5 ± 9.5 cc, range 2–40 cc. One aliquot was used to estimate cell counts and the remaining volume was centrifuged at 2000 g for 10 minutes. All samples were stored at −80°C.

### 2.2. Measurements

HA was determined by an enzyme-linked binding protein assay (Corgenix Inc, CO, USA). The total amount of TIMP-1, MMP-1, MMP-13, and MMP-3 was measured by the corresponding one step sandwich enzyme immunoassay (EIA) from Amersham Biosciences (UK, Buckinghamshire, England) and BioSource International, USA (MMP-3). Enzyme-linked immunosorbent assay (ELISA) kits were used for determining PDGF-AB, VEGF, HGF, IGF-I, and TGF-*β*1 concentrations (R&D Systems, Abingdon, UK).

### 2.3. Statistical Analysis

Results are presented as median and arithmetic mean ± standard deviations. The Pearson coefficient was used to evaluate the associations between plasma and SF biomarker concentrations. Factor analysis by the principal component (PCA) method was carried out to determine associations between molecular markers and reduce the data of the biochemical markers that are correlated. Components with Eigen values >1 were extracted. The interpretability of these markers was examined after applying a Varimax rotation with Kaiser Normalization. Further analysis of parametric samples was performed using the General Linear Method (GLM) approach to ANCOVA. Each principal factor representing combined biomarkers was used as a dependent variable; the Ahlbäck score in addition to sex was used as an independent variable; age and BMI were entered as covariables. Statistical analyses were performed using SPSS version 15.0 (SPSS, Chicago, IL, USA).

## 3. Results

The mean age of the participants was 66 ± 11 years (range 44–88) and 57% were females. Subjects had an average BMI of 29.07 ± 4.12 kg/m^2^ (range 22–40). The right knee was affected in 26 patients and the left in 27 while 10 patients had both knees affected. Of the 73 knee radiographs evaluated according to Ahlbäck classification: eleven (15%) graded Ahlbäck I, 22 (30%) graded Ahlbäck II, 27 (37%) graded Ahlbäck III, and 13 (18%) graded Ahlbäck IV.

Table 1 shows the biomarker concentrations in both plasma releasate and synovial fluid.

A marked degree of correlation (Pearson product moment correlation coefficient, *r* > 0.5) was observed for plasmatic IGF-I with IGF-I in synovial fluid (*r* > 0.577, *P* = .000) but not for other GFs, suggesting local synthesis of these factors and/or variable clearance kinetics.

Marked correlations were observed for platelet-secreted growth factors (TGF-*β*, PDGF, HGF, and VEGF) within plasma releasates (data not shown) while a moderate degree of correlation (*r* > 0.3) was observed between TGF-*β* and VEGF (0.340, *P* = .007) and between VEGF and HGF (0.265, *P* = .035) within the synovium.

The levels of plasmatic but not synovial HA associated with IGF-I in both fluids, with Pearson coefficients of −0.472, *P* = .000 (synovial fluid) and −0.415, *P* = .001 (plasma).

### 3.1. Associations of Biochemical Markers by the Principal Component Analysis: Relationship to Radiographic Severity

#### 3.1.1. Plasma Releasates

The correlated biomarkers were reduced to four independent factors explaining 70% of the total cumulative variance (Table 2). The platelet count in PRGF, PDGF-AB, and TGF-*β* 1 was loaded together in the first factor accounted for 24.79% of the variance. The second factor (IGF-I and HA) explains 15.91% of the variance. The third and fourth factors are represented by typical biomarkers of the catabolic balance, MMP-1/TIMP-2 and MMP-3/TIMP-1, and accounted for 14.97% and 14.68% of the variance, respectively. To test our hypothesis that the radiographic status entails different biomarker levels we used ANCOVA. In ANCOVA analyses we ascertained that the first (PDGF-AB and TGF-*β*) and the second (IGF-I and HA) factors representing the combined biomarkers are relevant to radiographic severity (*P* = .005 and *P* = .002, resp.).

#### 3.1.2. Synovial Fluid

The correlated biomarkers in synovial fluid were reduced to three factors; these together explain 51.84% of the total cumulative variance. The first factor comprised IGF-I and TIMP-2 and explains 20.4% of the variance. Angiogenic signaling factors (TGF-*β*1, VEGF, and HGF) accounted for 17% of the variance. HA and MMP-3 segregated in the third factor explained 14% of the variance. Any of these factors showed a significant connection to Ahlbäck grading.

## 4. Discussion

In the present study, we have compared growth factor contents (PDGF-AB, TGF-*β*1, IGF-I, VEGF, and HGF) in synovial fluid and platelet-rich plasma releasate from OA patients. The rationale for measuring GFs in OA stems from the original therapeutic option, presently under investigation, that is based on the intra-articular application of autologous PRGF [7]. Initially, we have compared the levels of GFs in PRGF and synovial fluid in OA to clarify the rationale of our hypothesis. Platelets contain high levels of TGF-*β* and PDGF, which may allow the possibility of using them as a vehicle for GF supplementation within the capsular joint [8]. Additional research in animal models indicates that TGF-*β* is crucial for cartilage maintenance and a deficiency results in OA-like changes [9], although this issue has not been confirmed in humans.

We have also examined a further group of molecules including HA, MMP-1, MMP-3, MMP-13, TIMP-1, and TIMP-2. Together these molecules have been listed as investigative and/or burden biomarkers according to the BIPED terminology [10]. Every single one may be representative of specific molecular mechanisms primarily involving synovial turnover, angiogenic signaling, and metabolic conditions in OA.

Because analyses of single molecules do not reflect the complexity of disease progression, a multivariate approach is required to better illustrate the complex dynamic networks that participate in the disease. In the present study, blood biomarkers were investigated in PRGF releasate as an alternative to serum. Both are fluid components that remain after the clotting process of plasma or full blood is completed. The former may be better in the study of PDGF and TGF-*β* since it does not contain leukocytes, improving homogeneity of the fluid and reducing variability. The principal component analysis in this fluid segregated (i) platelet-secreted factors possibly associated to angiogenesis (PDGF-AB and TGF-*β*), (ii) HA and IGF-I likely related to synovium turnover and cartilage or bone metabolism, (iii) MMP-1/TIMP2, and (iv) MMP-3/TIMP-1, which may reflect the catabolic status of the joint. Among the principal factors found in plasma, TGF-*β*1 and PDGF showed the most consistent association with OA severity. An association of serum TGF-*β*1 to radiographic severity has also been reported previously, although those samples were collected after 12 hours of daily activities [5].

Another finding showed a significant connection of HA and IGF-I to radiographic severity. HA has been previously associated with morphological progression of knee OA [11], whereas a systemic role for growth hormone and IGF-I has been previously described in the pathogenesis and progression of OA [12]. Despite these significant findings there are some caveats in the present study. First, plasma and synovial fluid results did not correlate. In fact, to truly understand the interactions and influence of food intake, circadian and activity-related variations in biomarker concentrations could help in defining more precisely the usefulness of these biomarkers and the most appropriate body fluid for analyses.

In contrast to current agreement of the great potential value of biomarker assessment in SF, we have only found a single component with clear biological interpretation, namely, the association of TGF-*β*1, HGF, and VEGF, which may reflect angiogenesis in the synovium. It is difficult to determine why SF biomarkers did not show any association to OA severity. It is possible that this failure may reflect the limited value of standard radiography. In addition, rapid changes in the joint in response to local perturbations along with the rapid turnover of synovial fluid and variations in the efficiency of clearance from the joint compartment may increase the inconsistency in synovial fluid measurements.

## Figures and Tables

**Table 1:** Levels of hyaluronic acid (HA), matrix metalloproteases (MMP-1, MMP-3, and MMP-13), tissue inhibitors of metalloproteases (TIMP-1 and TIMP-2), platelet-derived growth factor (PDGF-AB), transformed growth factor (TGF-beta), vascular endothelial growth factor (VEGF), hepatocyte growth factor (HGF), and insulin-like growth factor (IGF-I) were measured on knee synovial fluid and in platelet-rich plasma (PRP) releasate at a single point in time. Results are shown as mean ± SD, (median), and range (ng-pg/mL). ND not detected. *MMP-13 was measurable only in 13 of the 63 patients.

	PRGF	Synovial fluid
Leukocyte count	ND	<400/*μ*L
Platelet (x10^6^) /mL	385 ± 133	ND
	(356) 217 − 690	
PDGF-AB (ng/mL)	15.33 ± 7.48	ND
	(14.19) 3600 − 46725	
TGF-beta1 (ng/mL)	27.02 ± 11.17	0.75 ± 0.56
	(24.75) 8.39 − 57.55	(0.62) 0.24 − 3.46
VEGF (pg/mL)	200 ± 142	993 ± 533
	(169) 10 − 681	(857) 304 − 2544
HGF (pg/mL)	472 ± 210	672 ± 445
	(413) 56 − 1115	(592) 212 − 3768
IGF-I (ng/mL)	56.0 ± 21.0	51.5 ± 20.0
	(53.4) 20.0 − 117.0	(53.0) 6 − 100
HA (*μ*g/mL)	72 ± 66	1452 ± 694
	(50) 4 − 367	(1385) 363 − 3625
TIMP-1 (ng/mL)	48.6 ± 11.5	868.6 ± 511.6
	(46.0) 34.0 − 86.0	(720.0) 51 − 3218
TIMP-2 (ng/mL)	53.0 ± 24.8	120.2 ± 47.0
	(59.0) 3 − 88	(117.0) 32 − 220
MMP-1 (ng/mL)	2.55 ± 1.12	24.7 ± 40.6
	(2.45) 0.3 − 6.2	(13.0) 1.0 − 250.0
MMP-3 (ng/mL)	7.17 ± 3.69	571 ± 494
	(6.43) 1.96 − 27.18	(466) 25 − 2056
MMP-13* (ng/mL)	ND	0.056 ± 0.024
		(0.054) 0.033 − 0.119

**Table 2:** Principal component analysis coefficients of independent molecular marker factors in plasma from 63 patients with knee OA. Molecular markers were grouped into factors of related measures by principal component analysis using a Varimax rotation with Kaiser Normalization. Components with Eigen values >1 were extracted. Primary components of each factor are shown in bold type.

Biomarker	Factor			
	1	2	3	4
Platelet count	**0.846**	−0.255	0.106	0.061
PDGF-AB	**0.841**	0.183	−0.186	0.177
TGF-beta1	**0.853**	−0.294	0.024	0.164
VEGF	0.599	0.381	0.354	−0.069
HGF	0.293	0.356	−0.489	0.505
MMP-1	0.112	−0.011	**0.685**	0.091
TIMP-2	−0.037	−0.014	**0.828**	0.213
MMP-3	−0.025	−0.058	0.138	**0.832**
TIMP-1	0.318	0.127	0.246	**0.726**
IGF-I	0.020	**−0.821**	0.054	0.044
HA	−0.131	**0.774**	−0.004	0.137
